# ACCELERATE-BASSO: early experiences and emerging best practices for ontology development in behavioral and social science research

**DOI:** 10.1186/s13326-026-00363-8

**Published:** 2026-07-14

**Authors:** Yue Yu, Lu Kang, Susan Michie, William D. Duncan, John Beverley, Fang Li, Daniel W. McNeil, Nan Huo, HaiFang Li, Matthew Horridge, Mark A. Musen, Jiang Bian, Cui Tao

**Affiliations:** 1 Department of Artificial Intelligence and Informatics, Mayo Clinic, Jacksonville, FL USA; 2 UCL Centre for Behaviour Change, University College London, London, UK; 3 Department of Community Dentistry and Behavioral Science, College of Dentistry, University of Florida, Gainesville, FL USA; 4 Department of Philosophy, University at Buffalo, Buffalo, NY USA; 5 Division of Computational Medicine, Stanford University School of Medicine, Stanford, CA USA; 6 Department of Biostatistics and Health Data Science, Indiana University, Indianapolis, IN USA; 7 Center for Biomedical Informatics, Regenstrief Institute, Indianapolis, IN USA

**Keywords:** Behavioral and social science, Ontology, Best practices, Ontology engineering, Ontologies, Knowledge representation

## Abstract

**Background:**

Behavioral and social science research (BSSR) is essential for understanding human behavior and informing interventions, policy, and health outcomes; however, such research faces persistent challenges related to fragmented knowledge, imprecise and inconsistent terminology, and limited interoperability across studies. Ontologies provide a promising solution by enabling standardized, machine-readable representations of concepts and relationships to support data integration, knowledge synthesis, and reproducibility.

**Results:**

We present an initial overview of the ACCELERATE-BASSO Research Network, an NIH-supported consortium established to advance BSSR through ontology-driven approaches. Specifically, we describe the role of the Dissemination and Coordination Center (DCC) and the Best Practices Working Group (BPWG) in supporting a network of projects focusing on accelerating BSSR through ontology development and use. Based on the consortium’s early experiences, we summarize emerging ontology-driven practices, current interoperability efforts, and common methodological considerations identified across the participating projects. Rather than proposing a formal consensus framework, this work provides an initial consortium perspective on current ontology development activities and shared lessons learned. We also discuss the potential role of ontologies in supporting AI-related applications in BSSR.

**Conclusions:**

This paper provides an initial consortium perspective on ontology-driven approaches for advancing BSSR. By synthesizing early experiences, emerging practices, and shared challenges across multiple projects, it offers insights to inform future ontology development, interoperability, and community consensus while laying the foundation for more systematic evaluation and broader adoption within the BSSR community.

## Introduction

Behavioral and social science research (BSSR) aims to understand human and animal behavior across the lifespan and at multiple levels, encompassing diverse domains such as psychology, sociology, and cultural studies as well as complex challenges faced in modern society, including political, economic, and technological dynamics [1]. Findings from BSSR inform the diagnosis and treatment of mental disorders, guide policy development, and enhance understanding of individual- and population-level behavior and its relationship to broader social and environmental contexts [2]. The increasing emphasis on innovation in the behavioral sciences has led to a proliferation of theories, constructs, and measurement approaches, which has complicated the coherent development and application of knowledge. Consequently, BSSR faces significant challenges in precisely representing, organizing, synthesizing, applying, and generalizing its rapidly expanding body of research [2]. Despite the growing volume of scientific output, incompatible terminologies, heterogeneous classification systems, and diverse measurement practices hinder the integration of findings across studies [3, 4]. Researchers and practitioners also face difficulties in identifying relevant evidence, as well as in systematically testing, reproducing, synthesizing, and applying research outcomes, leading to inefficiencies in knowledge accumulation [5, 6]. Furthermore, unclear relationships between constructs and their measures, along with variability in study contexts and populations, limit the generalizability of findings [7]. These issues contribute to a widening gap between the generation of knowledge and its effective application in practice, ultimately constraining the impact of BSSR.

Ontologies are formal machine-readable representations of entities and relationships among them, designed to address interoperability problems and improve data quality within and across domains [8, 9]. In BSSR, this structured representation addresses key challenges such as imprecise and inconsistent terminology, fragmented classifications, and incompatible data by clarifying what phenomena are being studied and how they are related [6, 10]. Additionally, ontologies enhance information retrieval, support computational tools and automated reasoning, and provide infrastructure for organizing large-scale knowledge bases [11]. By enabling precise communication, ontologies support the consistent definition and measurement of constructs, facilitate data integration and sharing across studies, and allow researchers to compare and synthesize findings more effectively [12]. Through these mechanisms, ontologies can improve the reproducibility, generalizability, and accessibility of research, ultimately accelerating scientific discovery and enabling more effective translation of BSSR findings into policy and practice.

In the era of artificial intelligence (AI), ontologies play an increasingly critical role in enabling BSSR to fully leverage data-driven methods. As AI and machine learning models often depend on large, well-structured, and semantically consistent datasets, ontologies may facilitate data standardization, integration, and semantic interoperability, thereby supporting AI-related applications in BSSR [13]. However, the heterogeneity and ambiguity of behavioral and social science data often limit their effectiveness. Ontologies can provide semantic backbones that facilitate the standardization of constructs, harmonization of diverse data sources, and the encoding domain knowledge in a machine-readable form, thereby improving data quality and interoperability for AI applications [14]. In BSSR, the adoption of ontology-driven best practices—such as the use of shared vocabularies, standardized annotation protocols, and reusable ontology design patterns—can promote consistency and scalability across AI studies [15]. Moreover, ontology-guided data modeling can enhance the interpretability and transparency of AI models by linking features to well-defined concepts and relationships, facilitating explainable and trustworthy AI [16]. By supporting automated reasoning, knowledge integration, and scalable data annotation, ontologies—when coupled with well-defined best practices—enable more robust, reproducible, and generalizable AI-driven discoveries, ultimately accelerating the translation of BSSR insights into real-world applications.

## ACCELERATE-BASSO and the best practices working group

To accelerate BSSR through ontology development and use, the National Institutes of Health (NIH) has supported the ACCELERATE-BASSO (BehAvioral and Social Science through Ontology, BASSO) Research Network [17] since 2024. The project aims to address challenges in representing, integrating and standardizing knowledge across its complex and multidisciplinary landscape of BRRS. The ACCELERATE-BASSO Research Network currently includes four research projects alongside a Dissemination and Coordination Center (DCC). The objective of the DCC is to facilitate collaboration and cross-project learning, provide ontology-related technical and informatics expertise, promote the dissemination of resources and training, and engage stakeholders to advance the development and adoption of BSSR ontology tools and best practices.

As one of the core components of the ACCELERATE-BASSO project, the Best Practices Working Group (BPWG) aims to advance the consistent development and application of ontologies across the ACCELERATE-BASSO Research Network, and beyond. The Best Practices Working Group focuses on creating resources to support a shared framework for ontology development and use, guiding the adoption of tools and methodologies across projects, and providing ontology-related technical, computational, and informatics expertise. It aims to promote standardized, scalable, and interoperable ontology practices that support cross-project integration and accelerate advances in BSSR. In this paper, we present our current experiences and demonstrate how ontology-driven best practices can facilitate BSSR across and beyond the Network, particularly in the context of the rapid growth of AI.

As the ACCELERATE-BASSO Research Network is currently in the early stage of development, the primary objective of this paper is to introduce the background, current activities, early experiences, and significance of the Network in advancing BSSR through ontology-driven approaches. Specifically, this manuscript summarizes ongoing ontology development efforts across the network and highlights emerging best practices identified during the initial phase of the project. Rather than presenting a formal consensus standard or hypothesis-driven experimental study, this work aims to synthesize community-informed practices and recurring challenges relevant to advancing social and behavioral ontology development within translational research ecosystems.

## Ontology-driven best practices across ACCELERATE-BASSO research network projects

The ACCELERATE-BASSO Research Network currently includes four research projects, each focusing on distinct domains within BSSR and developing domain-specific ontologies to address unique challenges in their respective fields. Table 1 summarizes these projects and their associated ontologies within the ACCELERATE-BASSO Research Network. In the following subsections, we describe the ontology-related needs and gaps within each BSSR domain, provide an overview of project aims and current status, and present emerging best practices across these projects.

**Table 1:** ACCELERATE-BASSO research network project

BASSO Project	Associated Ontology Products
Advancing Prevention Research In Cancer through Ontology Tools Project (APRICOT)	Behavior Change Intervention Ontology (BCIO)
Standardizing and Harmonizing Behavioral and Social Science Research Factors in Alzheimer’s Disease through Ontology-Based Approaches	Behavioral Social Data and Knowledge Ontology for ADRD (BSO-AD)
Developing an Ontology for Dental Care-Related Fear and Anxiety: Toward an Understanding of Problems in Dental Care Utilization	Ontology of Dental care-related Fear, and Anxiety, and/or Phobia (ODFA)
Promoting Health Aging through Semantic Enrichment of Solitude Research (PHASES)	The Solitude Ontology and the Gerotranscendence Ontology (SOLO and GERO)

### APRICOT

BSSR lacks a shared, precise terminology based on a semantically coherent model of the domain. This has led to inconsistent reporting, fragmented datasets, and unnecessarily limited ability to discover evidence and to conduct evidence synthesis and cross-study integration [18–20]. There is also a need for more precise specification of behavior change theories, interventions, their mechanisms, contexts and methodologies [21, 22]. A stronger infrastructure is needed to address these limitations in a computationally accessible way [6, 23]. The APRICOT project (2024–2029), in extending and improving the Behavior Change Intervention Ontology (BCIO), and in developing associated tools and resources, covers the full gamut of BSSR [24–26].

Building on the Human Behaviour Change Project (2017–2023), the APRICOT project, a collaboration of behavioral scientists, data scientists, and ontologists, aims to accelerate data integration and knowledge accumulation in the behavioral and social sciences [24, 25]. The project has five aims: (1) improving the range and precision of ontological terms; (2) developing tools and resources for creating and maintaining ontologies; (3) developing ontology matching and alignment tools; (4) improving protocol and report writing and annotation tools; and (5) building a global Community of Practice of BSSR ontology users.

A range of tools and resources have been developed and are currently operational to support ontology-driven BSSR, including online platforms for BCIO search [27] and visualization [28], protocol authoring and annotation tools [29], behavior change technique–mechanism of action mapping tools [30, 31], and databases for exploring behavior change theories [32]. Additional resources include tools for ontology-based intervention development, data schemas for ontology-based annotation of documents and datasets [33, 34], and a Project Gateway hosted on Wellcome Open Research [35]. Furthermore, APRICOT is actively harmonizing the BCIO with the Ontology for Modeling and Representation of Social Entities (OMRSE), incorporating key social determinants such as income, education, and facilities, while also fostering a Community of Practice and delivering webinars and training programs to build capacity and promote adoption of BSSR ontologies [36].

The APRICOT project has identified several key best practices for advancing ontology-driven BSSR. First, generalizing ontology-based annotation methods developed for one behavior domain (smoking cessation) to others (currently, 24-hour movement behaviors, including physical activity, sedentary behavior and sleep). This supports scalable, cross-domain evidence synthesis. Second, mapping behavior change techniques to mechanisms of action using a formal ontology enables clearer hypotheses about how interventions have their effects, more rigorous testing of theory, and more accurate reporting of how interventions work i.e. their mechanisms of action. Third, Implementing FAIR (Findable, Accessible, Interoperable, Reusable) principles [37] from the outset of ontology development is essential to ensuring tools are adopted and have real-world impact. Finally, building a Community of Practice and the Behavioural and Social Sciences Ontology (BSSO) Foundry [38], alongside technical tools to promote access of relevant ontologies to behavioural and social science researchers, collaboration and co-creation, user engagement and sustained uptake across disciplines and countries.

### BSO-AD

BSSR factors play a pivotal role in shaping the risk, development, and outcomes of Alzheimer’s disease and related dementias (ADRD) across populations [39]. Integrating BSSR knowledge into ADRD prevention and intervention frameworks is critical for informing the development of targeted health policies [40, 41]. However, systematic integration remains a substantial challenge due to source heterogeneity and representation variability, necessitating a robust, interoperable ontological infrastructure. Compounding this challenge, existing ontologies remain largely siloed, focusing on either BSSR or ADRD in isolation, creating a critical gap in unified, cross-domain knowledge representation. The Standardizing and Harmonizing Behavioral and Social Science Research Factors in Alzheimer’s Disease through Ontology-Based Approaches project aims to bridge the gap in ADRD-related BSSR data integration by standardizing representation, enabling efficient extraction, and fostering collaboration within the research community.

To this end, we have developed the Behavioral Social Data and Knowledge Ontology for ADRD (BSO-AD). The development process followed the best practices tailored to the specific needs, encompassing: (1) principled reuse and alignment with existing ontologies, such as reusing classes from Social Determinants of Health Ontology (SDoHO) [42], Drug Repurposing-Oriented Alzheimer’s Disease Ontology (DROADO) [43] and ICD-10-CM Z codes [44]; (2) ontology relationship development grounded in the Relation Ontology (RO) [45], while leveraging the Unified Medical Language System (UMLS) [46] relational hierarchy as a conceptual framework to inform domain-specific relationship modeling. Rather than importing UMLS relations directly, we used the top-level relation “associatedWith” as a conceptual scaffold to guide the design of object properties within BSO-AD. Wherever possible, object properties were aligned with RO, and new sub-properties were introduced only when no suitable RO relation existed. For example, “functionallyRelatedTo” and “temporallyRelatedTo” were modeled as sub-properties of “associatedWith”. In addition, under broader relation “affects”, BSO-AD introduced ADRD-specific sub-properties, including “affectsRiskOf”, “affectsDiseaseCourse”, “affectsDiseaseBurden”, and “affectsPopulationMetric” tailored to the ADRD-BSF context. (3) a logically coherent and extensible structure that balances hierarchical expressiveness with manageable complexity; (4) unambiguous and standardized naming conventions that deliberately avoid vague terms. For ICD-10-CM concepts labeled as “other” or “unspecified,” BSO-AD maps these concepts to their corresponding parent classes while preserving the original source terminology through annotations and provenance metadata. For example, “Z56.0 Unemployment, unspecified” is mapped to the class “Unemployment” and retained as a narrower source concept using the annotation property “skos: narrower: Z56.0”, together with source attribution and descriptive metadata. This approach promotes semantic clarity while maintaining traceability to the original source terminology; (5) iterative refinement through both expert-in-the-loop review and large language model (LLM)-assisted, data-driven assessment. Ongoing efforts are focused on developing a natural language processing (NLP) pipeline, harnessing state-of-the-art LLMs, for scalable entity extraction and normalization from electronic health records (EHRs) and literature. Collectively, these endeavors will advance the understanding of ADRD through the lens of BSSR and contribute to evidence-based care and support for affected individuals and their communities.

### ODFA

Dental care-related fear, anxiety, and phobia (DFA) broadly refer to negative emotional, behavioral, or physical responses that occur when thinking about or being a patient receiving professional dental care [47]. Currently, research and clinical work with high DFA patients is stymied by the absence of an agreed-upon and controlled vocabulary/ taxonomy. The scientific literature on DFA constructs is inconsistent and includes terminology that refers to only dental fear or dental anxiety; these terms are often used interchangeably. In the broader psychological literature, however, fear and anxiety are treated as separate constructs [48] having unique manifestations [49]. The lack of consistency/interoperability in healthcare communities in defining and classifying these phenomena has contributed to the current stalemate in scientific progress as it relates to understanding the etiology and implications of DFA.

The goal of the Ontology of Dental Care-Related Fear, Anxiety (ODFA) is to address shortcomings in DFA terminology and classification. This effort is aligned with BSSR theory and research more broadly but also is specific to recent calls in the oral health domain for an inclusive, comprehensive approach to behavioral and social determinants of health [50]. The ODFA will facilitate the development of tools and resources capable of enhancing our understanding of the individual, clinical, and population impacts of dental care-related fear and anxiety. Specifically, we aim to develop an ontology that has acceptance from the scientific and clinical communities and that represents the multitude of types of fear and/or anxiety experienced by individuals to predict the likelihood of dental care avoidance, including emergency-only care, delay of treatment, cancellation of dental care appointments, or not showing up for scheduled appointments.

The ODFA is developed as a subset [51] of the Oral Health and Disease Ontology (OHD) [52, 53]. This permits the ODFA to easily relate DFA terms to other terms in the dental care space, such as restorations and root canal therapy. Furthermore, since the OHD is a member of the Open Biological and Biomedical Ontology (OBO) Foundry [54], the ODFA can leverage terms from other OBO Foundry ontologies, such as the Gene Ontology [55]. Although ODFA exists as a subset of OHD, it is hosted on BioPortal [56] as a stand-alone ontology and is included with BioPortal’s Behaviorial Social Science Ontologies slice [57]. This enables the DFA community to more directly access DFA-relevant terms without having the navigate the complete OHD. Currently, the ODFA contains 237 classes and 396 logical axioms.

The development of the ODFA follows OBO Foundry principles [58], with several best practices proving particularly valuable for ensuring quality and interoperability. These include requiring each term to have a unique label; requiring each term to have a unique IRI in the form of an OBO Foundry permanent URL; requiring each term to have a textual definition; requiring that the ontology be openly available using a Creative Commons license such as CC-BY [59] or CC0 [60]. In addition, ODFA have automated our release and versioning process utilizing the ROBOT [61] and Ontology Development Kit [62] resources.

### PHASES

Behavioral and social science research on aging lacks a shared, semantically coherent framework for representing constructs such as solitude, social isolation, loneliness, self-transcendence, and gerotranscendence. This has limited cross-study comparison, evidence synthesis, and the identification of intervention targets, despite the clear public-health importance of these domains. In older adulthood especially, time alone increases substantially, yet being alone can reflect importantly different psychological and social phenomena, some associated with distress and mortality risk and others with calmness, meaning-making, and well-being [19, 20]. At the same time, research on gerotranscendence points to potentially salutary developmental shifts in perspective, coherence, and connection in later life, but findings remain fragmented because disciplines use different terminologies, theories, and measurement strategies [63]. PHASES addresses this gap by developing ontology-based infrastructure to clarify and align these constructs, thereby strengthening behavioral science on healthy aging and enabling more coherent data integration, knowledge discovery, and intervention development [22, 63].

PHASES [64] is developing two interoperable ontologies [65] - the Solitude Ontology (SOLO) and the Gerotranscendence Ontology (GERO) - to formalize the constructs, theories, measures, interventions, and outcomes relevant to healthy aging [63]. Grounded in the Behavior Change Intervention Ontology (BCIO), these ontologies extend an existing upper-level framework rather than introducing a new silo, thereby supporting interoperability with the broader behavioral-science ontology ecosystem [22, 63]. Development follows established ontology-engineering practices, including competency-question driven design, stakeholder engagement, reuse of existing ontology resources where possible, explicit textual definitions, and automated quality control through open CI/CD workflows using GitHub, ROBOT, SPARQL, and release automation [54, 61, 63]. Beyond ontology development, PHASES is building a portal through which researchers, clinicians, and trainees will be able to interact with semantically enriched solitude and gerotranscendence data using recommender and question-answer functionality. In this way, PHASES contributes not only conceptual clarification, but also practical infrastructure for navigating literatures, identifying research gaps, and supporting intervention-oriented behavioral science [63].

Several best practices emerging from PHASES are relevant across ACCELERATE-BASSO ontology projects in behavioral science. First, ontology development should begin with stakeholder-centered competency questions, so that ontologies are driven by scientifically meaningful questions rather than abstract representation alone [63]. Second, projects should extend and align with shared reference ontologies, especially BCIO and other BFO-conformant resources, to avoid ontology silos and promote reuse, interoperability, and cumulative knowledge integration across behavioral domains [22, 54, 63]. Third, ontologies should be developed as open, computable research infrastructure, with globally unique identifiers, textual definitions, versioning, transparent governance, and automated quality control [52, 59]. Fourth, ontology projects in BSSR should connect formal representations to usable discovery tools, including semantic search, recommender systems, structured dataset annotation, and ontology-guided question answering [22, 63, 66]. Finally, PHASES suggests the value of combining expert-led ontology engineering with computational methods for concept discovery and ontology population, including BERTopic-based approaches, while preserving expert review as the basis for scientific validity [67]. Together, these practices can help U01 projects make behavioral-science knowledge more interoperable, scalable, transparent, and actionable.

## Discussion

This paper reports current experiences of the ACCELERATE-BASSO Research Network and demonstrates how ontology-driven best practices can facilitate BSSR. This work highlights the value of consortium-based collaboration for advancing semantic representation and interoperability of social and behavioral factors across diverse research domains. Although the participating projects differ in scope, knowledge domains, and implementation goals, several recurring themes emerged across consortium activities, including the need for semantic harmonization, ontology reuse, contextual representation, interoperability across heterogeneous systems, and sustainable governance strategies. Collectively, these experiences illustrate the practical challenges and opportunities involved in developing computable representations for complex social and behavioral constructs within modern biomedical research environments. Accordingly, the recommendations and observations described in this paper are intended to provide practical guidance and cross-project insights rather than definitive or fully validated standards. Future work will focus on more systematic evaluation frameworks, broader community consensus processes, and quantitative assessments of ontology interoperability, reuse, and downstream translational impact.

A key insight from this work is that ontology development in BSSR must balance domain specificity with cross-domain interoperability. Each project addresses unique challenges within its respective sub-field—ranging across behavior change interventions, Alzheimer’s disease, dental anxiety/fear and healthy aging—yet all benefit from alignment with common design principles. Practices such as reuse of existing ontologies, adherence to OBO Foundry principles [58], and implementation of FAIR guidelines [37] provide a foundation for interoperability. The ACCELERATE-BASSO DCC and Best Practices Working Group play an active role in promoting interoperability across ontologies both within and beyond the Research Network. These efforts include coordinating collaboration across projects, identifying common themes and differences in ontology development, sharing emerging insights, and developing guidelines for ontology development within the BSSR community. For example, the Best Practices Working Group has been developing a community guideline outlining emerging best-practice considerations for ontology development, as well as a white paper on ontology interoperability. While that effort remains ongoing, the current manuscript focuses on representative consortium projects and highlights cross-cutting methodological considerations, implementation experiences, and practical lessons learned from multiple perspectives. Rather than presenting a formal consensus standard or hypothesis-driven experimental study, this work aims to synthesize community-informed practices and recurring challenges relevant to advancing social and behavioral ontology development within translational research ecosystems. Together, these activities help ensure that local innovations contribute to a broader, reusable, and interoperable infrastructure for BSSR.

To further systematize the ontology-driven best practices emerging across the ACCELERATE-BASSO Research Network, Table 2 summarizes how individual research projects address key methodological aspects, including ontology reuse and alignment, adherence to FAIR and OBO Foundry principles, interoperability efforts, governance and maintenance strategies, BSSR-specific challenges and practices, and AI-related integration. As the network is still in the early stage of development, this summary provides an initial overview of shared practices and ongoing methodological efforts across the projects.

**Table 2:** Summary of emerging ontology-driven best practices across ACCELERATE-BASSO research network projects

BASSO Project	APRICOT	BSO-AD	ODFA	PHASES
Development Principles (FAIR, OBO, etc.)	• Follows OBO Foundry principles such as the principles of openness, defined scope, unique and unambiguous names, OBO metadata standards, text definitions, versioning, active maintenance, responsiveness and collaboration.	• Systematic ontology reuse, unique and unambiguous naming conventions, semantic interoperability, and alignment with other FAIR and OBO Foundry principles.	• The follows OBO Foundry principles for naming conventions, IRIs, labels, and definitions.	• PHASES develops SOLO, GERO, and related knowledge graphs using open, standards-based ontology engineering practices. The project uses RDF/OWL, OBO-style principles, persistent identifiers, definitions, synonyms, provenance, creator information, citations, licensing metadata, and competency questions to guide development and evaluation.
Ontology Reuse	• Import relevant, existing classes and properties from quality ontologies • Publish ontologies in standard formats (OWL2) • Formal version control and change management	• Reuse of relevant classes and properties from SDoHO, DROADO, ICD-9, ICD-10, TEO, AD-Onto, etc.	• We reuse several OBO Foundry ontologies including GO, RO, OBI, OGMS, IDO, and BFO.	• PHASES reuses existing ontology content wherever possible before introducing new terms. SOLO and GERO extend from the Behavior Change Intervention Ontology and reuse relevant BFO-conformant and OBO resources, including terms from OBI, OMRSE, the Mental Functioning Ontology, NCIT, HPO, the Emotion Ontology, and related resources. Novel domain-specific content is introduced only where existing ontologies do not adequately cover solitude, gerotranscendence, or healthy aging constructs.
Interoperability Efforts	• Build & grow BSSO Foundry • Co-develop ontologies (joint meetings, regular review) • Prevent & remediate redundant classes • Reuse shared, formal relations (object properties) • Use BFO as top-level ontology to ensure conceptual clarity at the highest level	• Adoption of standard annotation properties (e.g., dc, skos, and standardized identifiers); • Reuse of object properties (e.g., UMLS semantic network).	• The ODFA uses standard metadata properties defined by the OBO Foundries principles.	• PHASES is designed to prevent new silos in behavioral, social, biomedical, and healthy-aging research. The project aligns SOLO and GERO with BFO, BCIO, OBO-style practices, and the NSF-funded Proto-OKN technical expectations so that PHASES knowledge graphs can support semantic mappings, federated SPARQL queries, and integration with other knowledge graphs relevant to healthy aging, biomedicine, public policy, economics, ecology, and related domains. Mappings to other ontologies are expected, and will Follow the methodology described here: https://www.nature.com/articles/s41597-025-04580-1 with the expectation that a good mapping will serve as a rigorous foundation for code that uses it.
Ontology Maintenance	• The ontologies are regularly updated through a release process. • A release date is added to each release. • Changes are tracked in version control and previous versions are available through GitHub history • Zenodo is also used for formal versioning and archiving.	• Dynamic updates, version control, and tracking of ontology revisions.	• The ODFA is developed as a subset of the OHD, which is maintained using GitHub. OHD terms are tagged with metadata identifying it as an ODFA term. When a release of the OHD is made, the ODFA subset is extracted and released as a standalone ontology on Bioportal (https://bioportal.bioontology.org/ontologies/EMRO*).*	• PHASES uses a public GitHub-based development workflow for version control, issue tracking, stakeholder feedback, quality control, and release management. Ontology updates are guided by competency questions from domain experts and checked using ROBOT, SPARQL quality-control queries, GitHub Actions, and CI/CD workflows before release. This supports transparent, maintainable, and reproducible development of the PHASES ontology and knowledge graph suite
BSSR-Specific Challenges	• Lack of a common, precise semantically coherent terminology and set of constructs for developing and reporting behavioral interventions, their mechanisms of action and their evaluation. • Lack of readily available and usable tools to support ontology-based annotation of documents and data sets for evidence synthesis in behavioral science. • Lack of readily available and usable tools to use ontologies to support behavioral intervention development. • Under-specified and weakly conceptualized theories and models.	• Underrepresentation of BSSR factors in conventional biomedical terminologies, posing challenges in concept normalization, granularity definition, and relationship design.	• Developing fear and anxiety terms is a challenging task. Fear and anxiety are complex psychophysical processes with diverse patient-specific manifestations, and they present in unique ways in the dental-care setting.	• PHASES addresses the fact that behavioral and social science research often involves ambiguous, theory-laden, and indirectly measured constructs. In the solitude and gerotranscendence literatures, terms such as solitude, loneliness, social isolation, self-transcendence, ego integrity, and gerotranscendence are often used differently across disciplines, instruments, and research traditions.
BSSR-Specific Solutions	• Enhancing and extending the Behavior Change Intervention Ontology. • Development of data schemas for ontologized annotation of study reports and data sets. • Developing the Paper and Protocol Authoring and Annotation Tool (PAT) to support fully ontologized study design, reporting and annotation. • Development of the Theory and Techniques Tool (TaTT) and the COM-B Assessment Criteria To Intervention Strategy (CACTIS) tool to support the use of ontologies in intervention development. • Development of an ontology-based modelling system to characterize, compare and integrate models of behavior change.	• Systematic conceptual organization and semantic modeling as well as explicit relationship representation through literature mining and domain expert consultation.	• We have developed the Emotion Response Ontology to represent fear and anxiety as well as the physiological, verbal, psychological, and cognitive processes involved in experiences of fear and anxiety.	• PHASES responds to these challenges by building formal, machine-interpretable representations of key constructs, measures, theories, interventions, behaviors, and outcomes. SOLO and GERO distinguish constructs from the instruments and datasets used to measure them, clarify relationships among overlapping terms, and use BFO/BCIO-aligned modeling to show where research communities converge or diverge in their use of terminology. Put another way, PHASES aims to describe the differences among subject matter expert constructs rigorously enough to leverage machines to highlight where such constructs overlap and differ. The goal being to provide researchers better defined targets for empirical exploration
Application Scenarios	• Semi-automated ontology-based annotation of components of study reports. • Use of interpretable machine learning to predict outcomes in novel behavior change scenarios using ontologized data sets. • Integrated Human AI Knowledge Use (HAIKU) system using the CACTIS tool to support development of behavioral intervention strategies.	• As a semantic backbone to support data harmonization; • NLP/LLM-based information extraction; • Knowledge graph construction.	• We are using the ODFA’s terms to represent the meanings of questions used by dental fear and anxiety assessment instruments. When the terms are mapped to instance-level data, we can use the ODFA’s semantics to query for relations in data.	• PHASES supports semantically enriched research on healthy aging by enabling cross-disciplinary data integration, competency-question answering, recommender systems, question-answer tools, and AI-assisted research exploration. Applications include the creation of an AI-supported chat feature which leverages ontological relationships to filter plausible hypotheses worth investigating from large datasets. These are presented to subject matter experts who decide whether and how such to test such hypotheses. • Additionally, researchers are presented with specific logical differences between different uses of the same construct in the literature. Here AI is used to connect well-known ontology and modeling tools into an orchestrated agile workflow, following the methodology described here: https://ieeexplore.ieee.org/abstract/document/11338852

Abbreviations: FAIR, Findable, Accessible, Interoperable, Reusable; OBO Foundry, Open Biological and Biomedical Ontology Foundry; OWL, Web Ontology Language; BSSO, Behavioural and Social Sciences Ontology; BFO, Basic Formal Ontology; PAT, Paper and Protocol Authoring and Annotation Tool; TaTT, Theory and Techniques Tool; CACTIS, COM-B Assessment Criteria To Intervention Strategy; HAIKU, Human AI Knowledge Use; SDoHO, Social Determinants of Health Ontology; DROADO, Drug Repurposing-Oriented Alzheimer Disease Ontology; ICD, International Classification of Diseases; TEO, time event ontology; UMLS, Unified Medical Language System; BSSR, Behavioral and social science research; NLP, natural language processing; LLM, large language model; IRI, Internationalized Resource Identifier; GO, Gene Ontology; RO, Relation Ontology; OBI, Ontology for Biomedical Investigations; OGMS, Ontology for General Medical Science; IDO, Infectious Disease Ontology; OHD, Oral Health and Disease Ontology; SOLO, Solitude Ontology; GERO, Gerotranscendence Ontology; RDF, Resource Description Framework; OMRSE, Ontology for Modeling and Representation of Social Entities; NCIT, National Cancer Institute Thesaurus; HPO, Human Phenotype Ontology; NSF, National Science Foundation; Proto-OKN, NSF Proto Open Knowledge Networks; ROBOT, ROBOT is an OBO Tool; SPARQL, SPARQL Protocol and RDF Query Language; CI/CD, Continuous Integration and Continuous Delivery/Development

Furthermore, the experiences across all projects demonstrate that ontologies can serve as a critical bridge between raw data and AI applications by enabling structured data annotation, supporting data harmonization, and providing semantic foundations for explainable and trustworthy AI models. Emerging best practices—such as ontology-based annotation pipelines, mapping of constructs to mechanisms, and integration with natural language processing tools—illustrate how ontologies can enhance both the scalability and interpretability of AI-driven research. Given the current developmental focus of our network, individual projects are actively developing domain-specific evaluation strategies and use cases to assess ontology utility, interoperability, reproducibility, and downstream AI-enabled applications. More detailed quantitative and qualitative assessments will be reported in future publications by the respective project teams.

In addition to the Best Practices Working Group, the ACCELERATE-BASSO initiative has established complementary working groups, such as the Resource Sharing Working Group, to support the dissemination of tools, resources, and findings to the broader BSSR community. These groups play an important role in fostering knowledge exchange and enhancing the visibility and accessibility of ontology-related outputs. As the initiative evolves, additional working groups may be formed to address emerging challenges and adapt to the changing needs of the BSSR landscape.

Despite these advances, several challenges remain. Ontology development in BSSR requires significant interdisciplinary collaboration, and the adoption of ontological methods can be hindered by limited familiarity among domain researchers and the perceived complexity of ontology engineering [68]. In addition, achieving consensus on definitions and classifications across diverse fields of BSSR is inherently difficult. Although the participating projects share a common commitment to ontology-driven BSSR, achieving interoperability across independently developed ontologies remains a significant challenge. The projects differ in scope, conceptual models, granularity, and intended applications, creating potential inconsistencies in how similar concepts, relations, and contextual factors are represented. Behavioral and social constructs are often defined differently across research communities, and equivalent concepts may be modeled at different levels of abstraction. Consequently, ontology alignment requires more than simple term mapping and may involve resolving conceptual differences, developing bridging axioms, harmonizing relation usage, and establishing shared modeling conventions. These challenges are further complicated by the context-dependent nature of many BSSR concepts, where meaning and relevance may vary across populations, settings, and research objectives. Beyond these challenges, a critical gap lies in the lack of well-defined methods and protocols for ontology development and alignment. For example, there is currently limited guidance on how to align overlapping ontologies, how to determine ownership of classes and relations across namespaces, and what level of semantic richness is appropriate for different use cases. Furthermore, distinctions between ontologies designed to represent subject matter expert (SME) knowledge and those intended to support SME-facing applications remain underexplored. At present, AI-related applications across the projects remain under active development and evaluation. In particular, future work is needed to assess whether and how ontologies improve information extraction accuracy, reduce ambiguity, enhance model generalizability, or support explainability artifacts that are meaningful to users. Sustainability and long-term governance also pose challenges, including the need for continuous updates, community engagement, and alignment with evolving standards and technologies. While networks such as ACCELERATE-BASSO aim to support coordination and knowledge sharing, addressing these methodological gaps will require the development of concrete, community-endorsed standards and protocols rather than increased coordination alone. Identifying and formalizing such best practices remains an important direction for future work.

Looking forward, continued efforts are needed to expand ontology coverage across underrepresented BSSR domains, enhance tools and resources for ontology development and application, and promote training and community engagement to support broader adoption. The integration of ontology frameworks with emerging AI and LLM technologies presents a promising direction for automating knowledge extraction, supporting data harmonization, and accelerating discovery. By combining domain expertise, standardized ontological frameworks, and advanced computational methods, the ACCELERATE-BASSO project provides a model for advancing BSSR toward a more interoperable, reproducible, and impactful scientific paradigm.

## Data Availability

No datasets were generated or analysed during the current study.

## Abbreviations

ADRD: Alzheimer’s disease and related dementias
AI: Artificial Intelligence
APRICOT: Advancing Prevention Research In Cancer through Ontology Tools Project
BASSO: BehAvioral and Social Science through Ontology
BCIO: Behavior Change Intervention Ontology
BFO: Basic Formal Ontology
BPWG: Best Practices Working Group
BSO-AD: Behavioral Social Data and Knowledge Ontology for ADRD
BSSO: Behavioural and Social Sciences Ontology
BSSR: Behavioral and social science research
CACTIS: COM-B Assessment Criteria To Intervention Strategy
CI/CD: Continuous Integration and Continuous Delivery/Development
DCC: Dissemination and Coordination Center
DFA: Dental care-related fear, anxiety, and phobia
DROADO: Drug Repurposing-Oriented Alzheimer’s Disease Ontology
EHRs: electronic health records
FAIR: Findable, Accessible, Interoperable, Reusable
GO: Gene Ontology
HAIKU: Human AI Knowledge Use
HPO: Human Phenotype Ontology
ICD: International Classification of Diseases
IDO: Infectious Disease Ontology
IRI: Internationalized Resource Identifier
LLM: large language model
NCIT: National Cancer Institute Thesaurus
NLP: natural language processing
NIH: National Institutes of Health
NSF: National Science Foundation
OBO Foundry: Open Biological and Biomedical Ontology Foundry
OBI: Ontology for Biomedical Investigations
ODFA: Ontology of Dental care-related Fear, and Anxiety, and/or Phobia
OGMS: Ontology for General Medical Science
OHD: Oral Health and Disease Ontology
OMRSE: Ontology for Modeling and Representation of Social Entities
OWL: Web Ontology Language
PAT: Paper and Protocol Authoring and Annotation Tool
PHASES: Promoting Health Aging through Semantic Enrichment of Solitude Research
Proto-OKN: NSF Proto Open Knowledge Networks
TaTT: Theory and Techniques Tool
RDF: Resource Description Framework
RO: Relation Ontology
ROBOT: ROBOT is an OBO Tool
SDoHO: Social Determinants of Health Ontology
SOLO and GERO: The Solitude Ontology and the Gerotranscendence Ontology
SPARQL: SPARQL Protocol and RDF Query Language
TEO: time event ontology
UMLS: Unified Medical Language System
